# Insights from lipidomics into the terminal maturation of circulating human reticulocytes

**DOI:** 10.1038/s41420-025-02318-x

**Published:** 2025-02-27

**Authors:** Giampaolo Minetti, Isabel Dorn, Harald Köfeler, Cesare Perotti, Lars Kaestner

**Affiliations:** 1 Department of Biology and Biotechnology “L. Spallanzani”, University of Pavia, Pavia, Italy; 2 Department of Blood Group Serology and Transfusion Medicine, Medical University of Graz, Graz, Austria; 3 Core Facility Mass Spectrometry, Medical University of Graz, Graz, Austria; 4 Servizio Immunoematologia e Medicina Trasfusionale, Fondazione IRCCS Policlinico San Matteo, Pavia, Italy; 5 Dynamics of Fluids, Experimental Physics, Saarland University, Saarbrücken, Germany; 6 Theoretical Medicine and Biosciences, Medical Faculty, Saarland University, Homburg, Germany

**Keywords:** Biochemistry, Cell biology

## Abstract

In the age of “omics”, lipidomics of erythropoiesis is still missing. How reticulocytes mature in the circulation into functional erythrocytes is also largely unknown. We have isolated here two populations of human circulating reticulocytes at different levels of maturation, and three subpopulations of erythrocytes of different age, and characterized the evolution of their lipidome. (Sphingomyelin+cholesterol) and partly phosphatidylethanolamine increase relative to total lipids, whereas phosphatidylcholine and phosphatidylserine decrease from immature reticulocytes to mature erythrocytes, at the same time as the surface area per cell decreases. The relative amounts of more than 70 phospholipid subclasses, based on the number of carbon atoms (12–24) and of double bonds (0–6) in the fatty acids linked to the phospholipid, also change in the process. As reticulocytes and erythrocytes cannot perform de-novo phospholipid synthesis, lipid remodeling likely requires selective removal of phospholipids from the membrane or their exchange with plasma or both, with the possible involvement of lipid transfer proteins such as VPS13A, which is expressed in reticulocytes and erythrocytes. These findings not only shed light on fundamental aspects of red blood cell physiology and erythropoiesis but also raise intriguing questions surrounding protein-lipid interactions, membrane architecture, and lipid trafficking mechanisms.

## Introduction

The process of terminal differentiation from reticulocytes to mature red blood cells (RBCs) in mammals has long been a subject of intense scrutiny since the inception of RBC research [1]. Recent years have seen a resurgence of interest in this area [2, 3], also aided by “omics” approaches [4–6] particularly in light of the potential for culturing human RBCs in vitro for transfusion purposes [7, 8]. While considerable knowledge has been amassed regarding the early phases of reticulocyte maturation within the bone marrow of adult mammals following enucleation of orthochromatic erythroblasts, the terminal development of circulating reticulocytes into mature RBCs remains elusive. The main problem was the availability of pure populations of normal, “non-stress” circulating reticulocytes at specific stages of differentiation [9, 10]. As a result, the issue as to whether peripheral circulating reticulocytes could mature into “normal” RBCs in vitro is controversial [11]. Secondly, characterization of terminal erythroid differentiation has been more recently conducted primarily through proteomics and transcriptomics approaches [12], with limited attention to lipids as essential membrane constituents. This occurred despite the fact that characterization of RBC lipids predated that of proteins, leading to seminal discoveries on membrane architecture in eukaryotic cells, such as the asymmetric transverse localization phospholipids in the two leaflets of the bilayer [13–15].

To address these challenges, we isolated subpopulations of circulating reticulocytes at various maturation stages in vivo, along with subpopulations of RBCs of different age from normal human donors and characterized their lipid content. Furthermore, in all the cell subpopulations under study and other blood cell types we conducted a semi-quantitative analysis of protein VPS13A, an ubiquitously expressed protein with the recently recognized role of a bridge-like lipid transfer protein that is missing in Chorea acanthocytosis patients [16].

## Results

### Cell size and shape

**Fig. 1:**
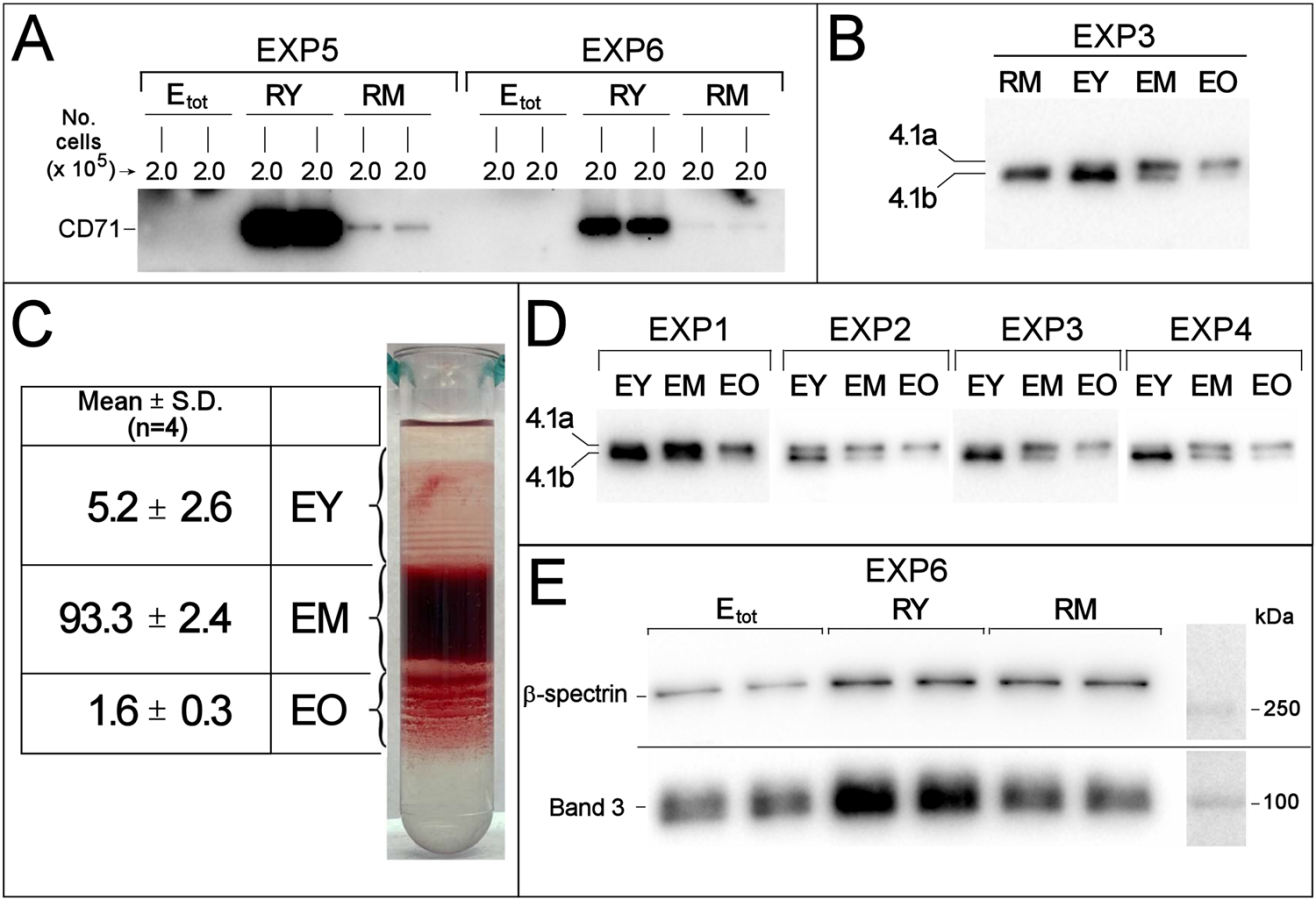
Characterization of the subpopulations of circulating reticulocytes and RBCs of different age subjected to the study. **A** Western blotting with anti CD71 of RY and RM reticulocytes and of total RBCs (E_tot_) from the corresponding donor in two representative experiments. For each sample, 2.0 × 10^5^ cells were loaded. The large differences in signal intensity between samples containing identical numbers of cells prevented quantification by densitometry of the bands. Instead, the relative differences in CD71 expression were evaluated by the procedure described in detail in the Supplementary material and Fig. S1A–C. **B** Protein 4.1 R Western blotting in reticulocytes and RBCs of different age. The blotting is representative of four independent experiments with identical results. RM, EY, EM, and EO were from the same donor. In each lane, 2.0 × 10^5^ cells were loaded. See Supplementary material and Fig. S1D. **C** Separation of RBCs according to density in self-forming Percoll^®^ gradients as shown for one tube representative of four independent experiments. The table gives the average cell recovery in the three RBC subpopulations, evaluated as % Hb (or number of cells) recovered in each fraction with respect to total Hb (or number of cells) loaded in the gradient, for four independent experiments with blood from four different donors. **D** Western blotting of protein 4.1 in density-separated RBC subpopulations. The relative increase in protein 4.1a/4.1b ratio from young to old RBCs confirms that density separation resulted in a separation of RBCs of different age. Each sample contained 2.0 × 10^5^ cells. **E** Band 3 and β-spectrin levels in RY, RM and E_tot_ evaluated by Western blotting. The same number of cells (2 × 10^5^) was loaded for each sample, in duplicate. The blot shown is representative of four independent experiments with similar results. See also Supplementary material and Fig. S1G.

**Fig. 2:**
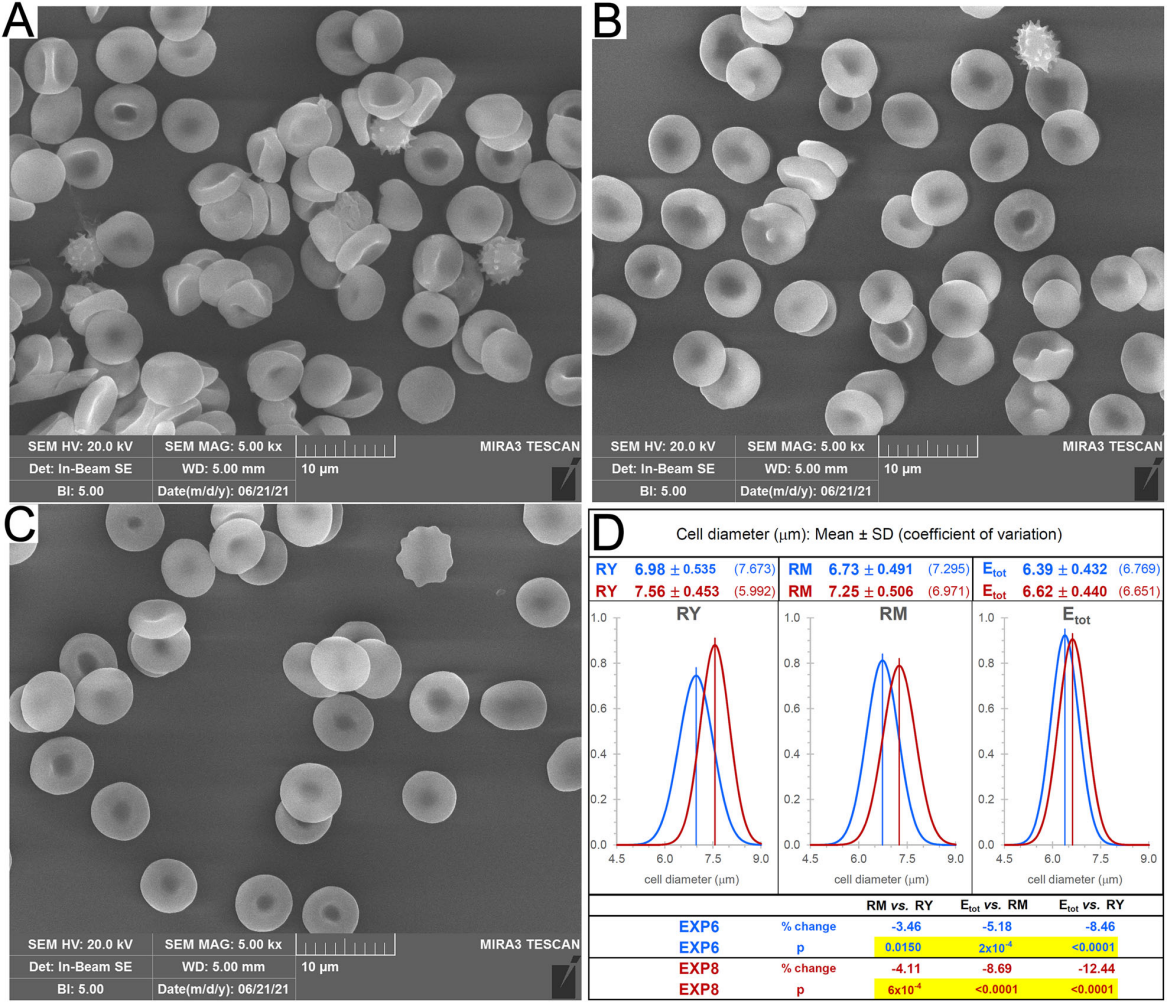
Characterization by microscopy of the morphology and size of the subpopulations of cells analysed in the study. Scanning electron microscopy of (**A**) young circulating reticulocytes, RY; (**B**) mature reticulocytes, RM; (**C**) RBCs from the total population of reticulocyte-depleted cell sample of the same donor, E_tot_. Images are representative of four independent experiments with similar results. **D** Diameter of RY, RM, and E_tot_. Unstained cells were fixed with glutaraldehyde [73], visualized in bright-field microscopy at 40× magnification and photographed with a digital camera. Thanks to a superimposed ocular micrometer, the diameter of 65 cells for each sample (RY, RM and E_tot_, the latter representing the total population of RBCs after isolation of reticulocytes) was manually measured in the digital image. The procedure was repeated with blood from two different donors. The measurements were normally distributed for each of the three samples (RY, RM, and E_tot_) from both donors, as verified by D’Agostino & Pearson test. The corresponding Gaussian curves were therefore plotted as displayed in the figure, with samples from donor of experiment 6 in blue and those from experiment 8 in dark red. The adjusted *p* values resulting from the analysis by one-way ANOVA and Tukey correction for multiple comparisons are reported and indicate that cell diameter decreases in a statistically significant manner as cells mature from RY to RM and from RM to E_tot_.

We have isolated, starting from a single sample of blood, two populations of CD71^+^ reticulocytes, RY and RM, representative of younger (≈0.1–0.2% of total blood cells) and more mature circulating reticulocytes (≈0.4–0.5% of total blood cells). Differences in the maturation stage were assessed by analysing the relative differences in CD71 expression: the levels of CD71 in RY were 20 to 30 times the levels in RM (Fig. 1A). Furthermore, the two populations of reticulocytes were free of contaminating mature RBCs (Fig. 1B). Additionally, from the reticulocyte-depleted population of RBCs we have separated subpopulations of young (EY), middle-age (EM) and old (EO) erythrocytes, amounting, respectively, to the following percentages with respect to total cells subjected to density gradient separation: 5.2 ± 2.6, 93.3 ± 2.4, and 1.6 ± 0.3 (mean ± S.D.; *n* = 4; Fig. 1C). Analysis of the protein 4.1a/4.1b ratio confirmed that the subpopulations of RBCs of different density also differed by cell age (Fig. 1D). When examined by scanning electron microscopy (SEM), almost all reticulocytes displayed the morphology of a biconcave discocyte, with a few misshaped cells, mostly present in the RY samples (Figs. 2A–C and S2), indicating that reticulocytes are almost indistinguishable from mature RBCs from the morphological point of view at stasis. The projected area diameter decreases by approximately 10% as immature reticulocytes (RY) mature to erythrocytes (E_tot_) (Fig. 2D).

Band 3 and spectrin, which are retained in the membrane of the maturing reticulocyte in the marrow and are not lost in the exosomes [17], display a decrease during the maturation of circulating reticulocytes (Fig. 1E). Conversely, the lipids which are characteristic of membrane rafts, sphingomyelin and cholesterol, and which have been described to be enriched in the exosomes released from maturing marrow reticulocytes [18], appear to be selectively retained in the membrane of reticulocytes maturing in the circulation, at the same time as their surface area decreases.

### Lipids

**Fig. 3:**
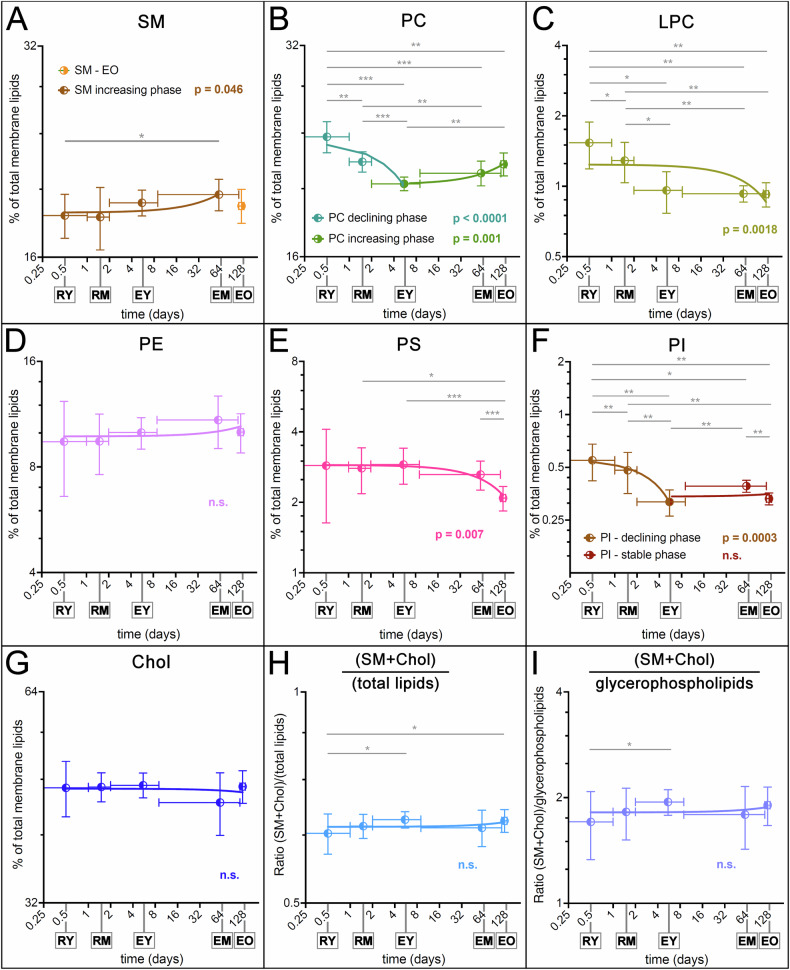
Quantitative changes in the main membrane lipids during maturation of reticulocytes and aging of RBCs in the circulation. Regression analysis of the changes in membrane lipid content during maturation of circulating reticulocytes and aging of RBCs. The original values, expressed as percent of a given lipid with respect to total lipids from the same sample (Panel **A**: SM; **B**: PC; **C**: LPC; **D**: PE; **E**: PS; **F**: PI; **G**: Chol) or the values for the ratios (SM + Chol) over total lipids (panel **H**) and (SM + Chol) over total glycerophospholipids (panel **I**), have been logarithmically transformed and plotted against the time, in days, also on a logarithmic axis, assuming an estimated cell age in the circulation of 12–24 h, 24–48 h, 1 week, 17 weeks, for RY, RM, EY, and EO, respectively (see also text). Data were interpolated by linear regression and the statistical significance of the slope being different from zero, i.e., indicating a change, is shown in colour. Statistical analysis of pairs of samples is also shown, when significant, as horizontal lines connecting the two samples under test. *N* = 7. *: *p* < 0.05; ** *p* < 0.001; *** *p* < 0.0001. The tabulated original values and their direct graphical rendering are shown in Table S3 and Fig. S3, respectively.

The availability of subpopulations of reticulocytes and RBCs of defined cell age allowed a regression analysis of the changes in lipid content versus time over the entire life span of the human RBC. Figure 3 shows the content of seven membrane lipids, as mol % of each species over total lipids, in the five cell populations under study (**tabulated values in** Table S3). A relative increase in cholesterol and SM and a decrease in PC and PS from reticulocytes to RBCs is observed, confirming our previous observations [19]. Starting from young reticulocytes (RY) the statistically significant relative increase in SM appears to plateau at the mature RBC stage (EM) (Fig. 3A). The opposite behaviour is displayed by PC, with a significant decrease from RY to EM. PC also displays an increase phase in the second half of RBC circulatory life, i.e., from EM to EO (Fig. 3B). LPC (Figs. 3C and S9), PS (Fig. 3E) and PI (Figs. 3F and S10) display a significant decrease all along reticulocyte maturation and RBC aging. PE (Fig. 3D) displays a relative increase, although not statistically significant, from reticulocyte to EM, followed by a decrease in EO (see also Table S3 and Fig. S3). Concerning cholesterol (Fig. 3G), although changes do not display statistical significance in any direction (contrary to the relative increase over total lipids previously reported by us [19]), when it is expressed as (cholesterol+SM) over total lipids (Fig. 3H) or total glycerophospholipids (Fig. 3I) a significant increase is again observed during maturation from reticulocyte to RBC.

### Phospholipid subclasses

When the different subclasses of a given phospholipid are analysed, they are seen to change, to variable extents, in relative abundance in the cell populations examined. Their changes should always be read in context with the underlying change in the relative amounts of that same phospholipid with respect to total lipids, be it a decrease or an increase (Fig. 3).

#### Phosphatidylcholine

**Fig. 4:**
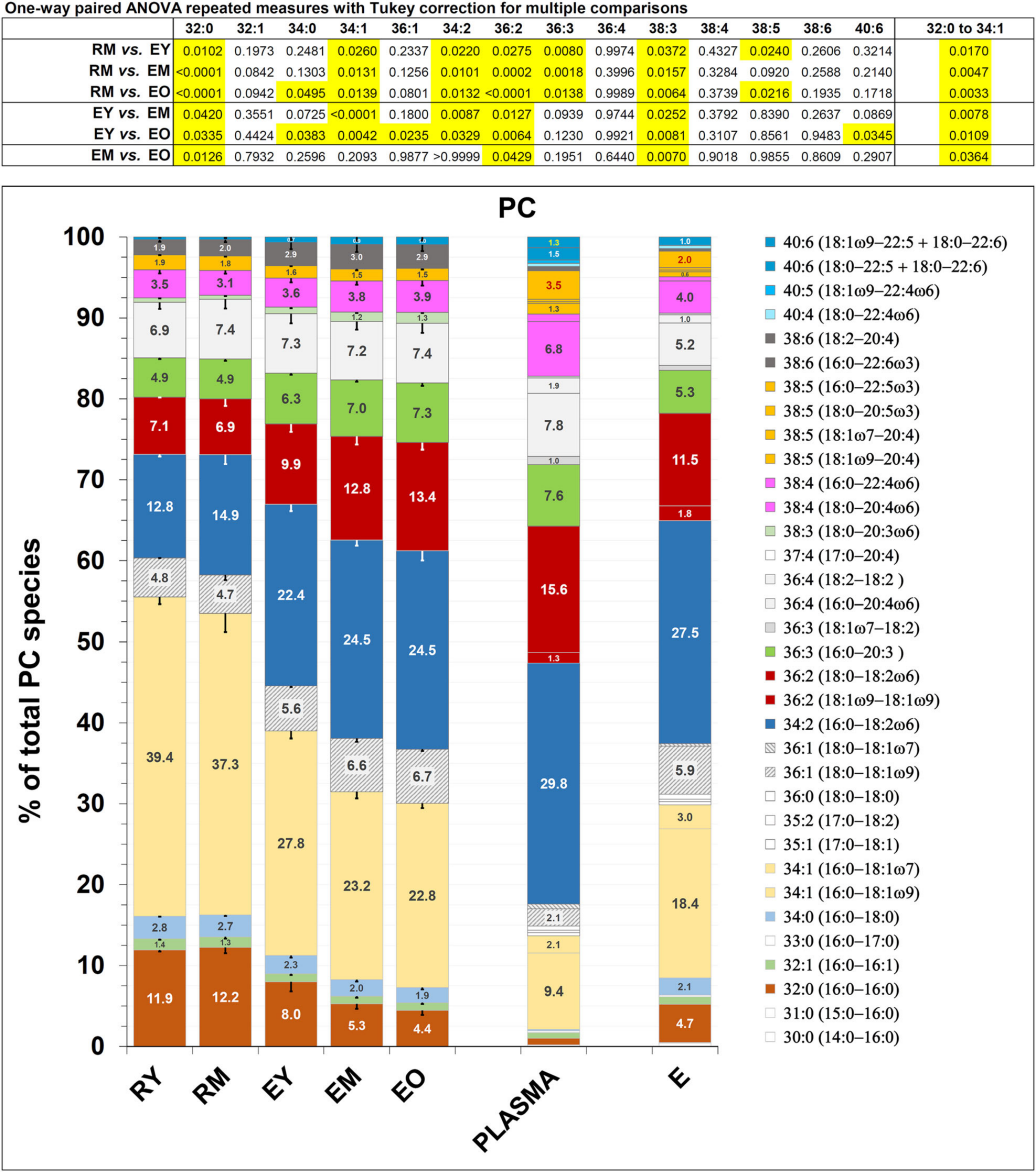
Diacyl PC subclasses in reticulocytes and RBCs. PC subclasses, as mol % of total PC in a given sample (as indicated by numbers inside the histogram bars) are presented with arbitrary colours that will be used consistently for the corresponding phospholipid subclass of diacyl PE, when present (Fig. 5). The PC composition of samples “PLASMA” and “E” (total population of RBCs) is taken from [20, 21], which was also used as a source for annotating the lipid subclasses obtained in our analysis. PC is ≈70% by weight of total lipids in plasma lipoproteins and ≈25 mole % of total phospholipids in RBCs [20, 44]. The PC composition of EM is virtually superimposable to that of the literature sample “E” [57]. Moreover, the PC subclasses composition of plasma and RBCs was already described in earlier studies [78], and it has been reported in a recent one [79] with virtually identical results (see also Fig. S5). The single saturated species (18:0/18:1) displaying a relative increase, instead of a decrease like all other saturated species (in bold in the legend to the right), is filled with dashed diagonal lines. Phospholipid subclasses were analysed in detail in a subset of 3 different donors. Error bars represent the standard deviation except for RY, where only two samples were available for analysis; here error bars represent the difference between the mean and the individual values. Differences between samples were statistically analysed by paired one-way ANOVA and Tukey correction for multiple comparisons resulting in the adjusted p values reported in the table on top of the graph, and highlighted in yellow where indicating statistically significant differences. See also Table S4 and Fig. S5.

Figure 4 shows such changes for PC subclasses (see also Table S4 and Fig. S5). The PC composition of RBCs taken from the literature [20] (“E” in Fig. 4) is practically superimposable to that of our sample of middle-age RBCs (EM). We show for the first time that analysis of phospholipid subclasses in the total population of RBCs yields an average value only, as the lipid composition varies widely depending on the age of the cell. A continuous remodelling of phospholipid subclasses occurs all along the maturation from RY to RM, then EY and during the maturation and ageing of RBCs. In the case of PC, that shows an initial decrease relative to total lipids and then an increase from EM to EO (Fig. 3B), individual PC subclasses change their relative amounts with a bimodal behaviour, all the way from RY to EO (Fig. 4). One group of PC subclasses, containing saturated acyl chains, decrease relative to total PC [dipalmitoyl-PC (16:0/16:0), 1-palmitoyl,2-stearoyl-PC (16:0/18:0) and 1-palmitoyl,2-oleoyl-PC (16:0/18:1)], while and a second group, containing unsaturated acyl chains, displays a relative increase. In Fig. 4, the composition in PC subclasses of plasma lipoproteins, (“PLASMA”) and total RBCs (E), taken from a literature dataset is also shown [20, 21]. Interestingly, as RY mature to EM, the relative amounts of a given PC subclass (with a few exceptions) appear to approach the content of that particular subclass in plasma.

#### Phosphatidylethanolamine

**Fig. 5:**
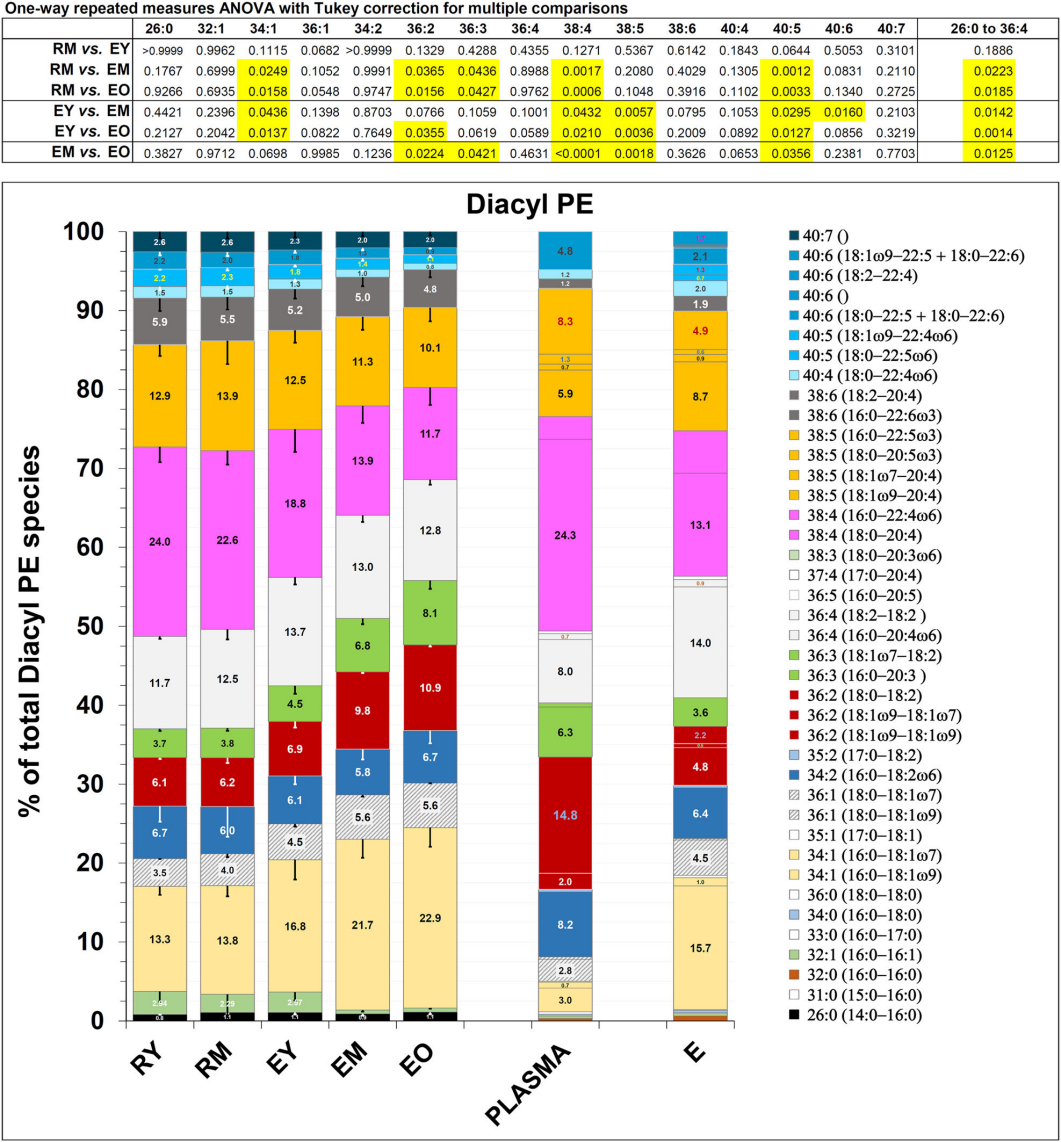
Subclasses of diacyl PE in reticulocytes and RBCs. Each subclass is given as mol % of total diacyl PE in a given sample. The diacyl composition of samples “PLASMA” and “E_tot_” (total population of RBCs) is taken from [20, 21], which was also used as a source for annotating the diacyl PE lipid subclasses obtained in our lipidomics analysis (see Materials and Methods). Diacyl PE subclasses of corresponding fatty acid composition as PC subclasses shown in Fig. 4 are shown in the same colour. Tabulated values can be found in Table S5. For error bars and statistical analysis, see the legend to the previous Figure.

Diacyl PE subclasses also appear to change according to a pattern. Again, this phospholipid’s subspecies can be divided in two groups. Those belonging to the first group display a relative increase with maturation, and contain relatively shorter and more saturated fatty acids (from 26:0 to 36:4). Those belonging to the second group contain longer and more unsaturated fatty acyl chains (from 38:4 to 40:7) (Fig. 5 and Table S5). Changes appear to be specular to those undergone by PC subclasses but, unlike for PC, PE subclasses do not give the impression of a tendency to equilibrate with the corresponding plasma diacyl PE species, as changes go in the opposite direction (with a few exceptions) with respect to the PE plasma composition.

#### Sphingomyelin

**Fig. 6:**
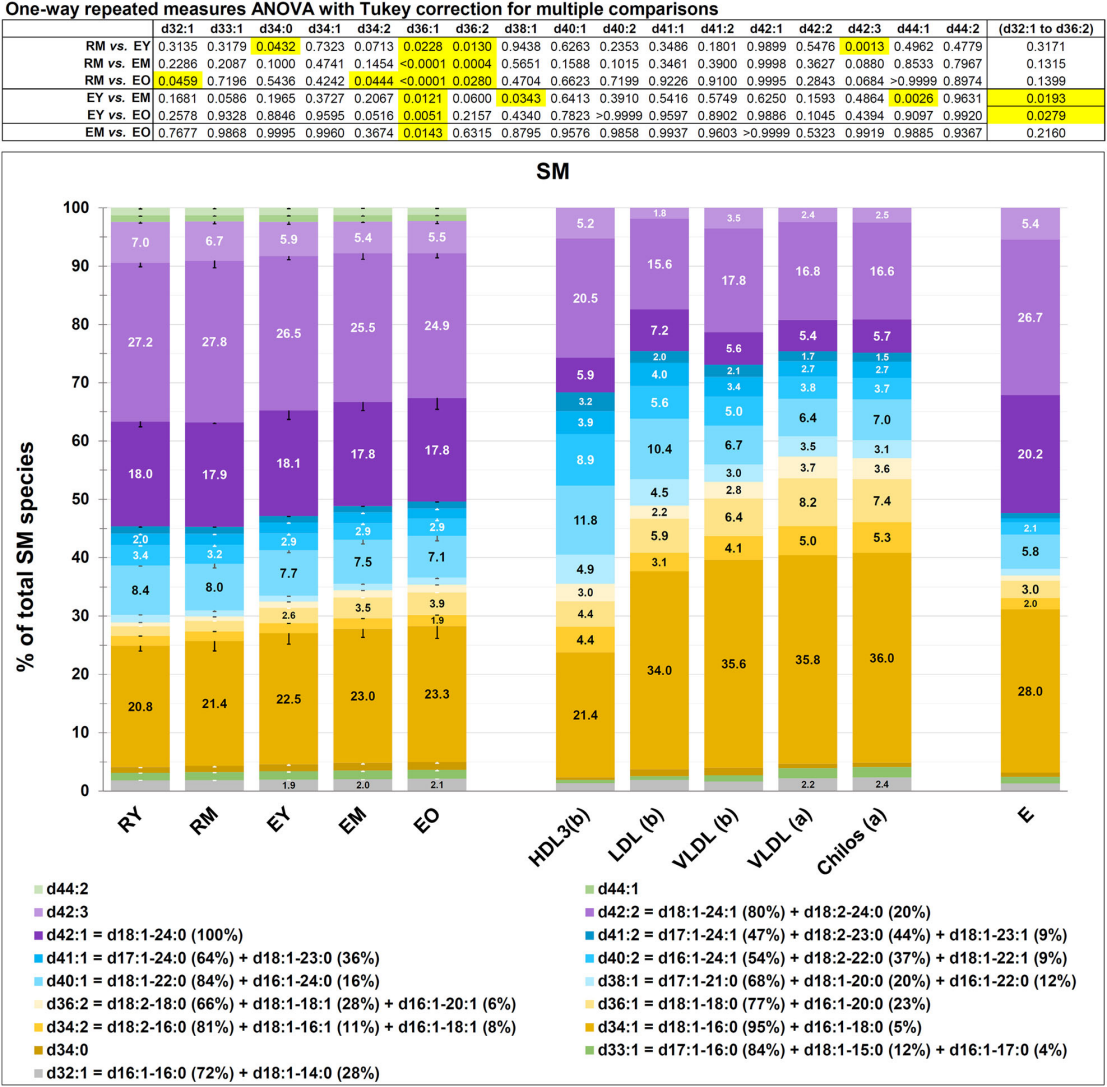
SM subclasses in reticulocytes and RBCs. Each subclass is given as mol % of total SM in a given sample, as indicated by numbers inside the histogram bars. The SM composition of “E_tot_” (total population of RBCs) is taken from [20], that of plasma lipoproteins from [21]. These two articles have been also used as a source for annotating the SM subclasses obtained in our lipidomics analysis. SM is ≈18% by weight of total lipids in plasma lipoproteins and ≈25% of total phospholipids in RBCs. (a) Postprandial plasma of a normolipemic subject; (b) fasting plasma from three different normolipemic subjects [21]. For error bars and statistics, see previous Figure legend. See also Table S6 and Figs. S6 and S7.

Changes in the relative abundance of various SM subclasses occur to a lesser extent than for PC, and continue to the stage of EO (Fig. 6) (see also Table S6 and Figs. S6–S8). In Fig. 6, the SM subspecies that are found in individual classes of plasma lipoproteins are also shown, taken from the literature [20, 21]. As for PC, most of the SM subclasses of the reticulocyte membrane give the impression of approaching the SM composition of plasma lipoproteins (except for the HDL family) as the reticulocyte matures. Furthermore, as with PC, a certain regularity of variation can be singled out. SM species carrying amidated fatty acids with less than 20 carbon atoms (from d32:1 to d36:2 included) display a relative increase in abundance, whereas those carrying a fatty acid with 20 or more carbon atoms decrease, pointing to a potential differential behaviour of inner *vs*. outer leaflet SM subclasses [22].

#### Phosphatidylserine

PS subclasses with the longest and more unsaturated fatty acids decrease more, relative to total PS species, at the same time that total PS undergoes a decrease with respect to total membrane lipids, from RY to EO (Fig. 3E, Table S3 and Figs. S3 and S8).

### Protein VPS13A in reticulocytes and RBCs

**Fig. 7:**
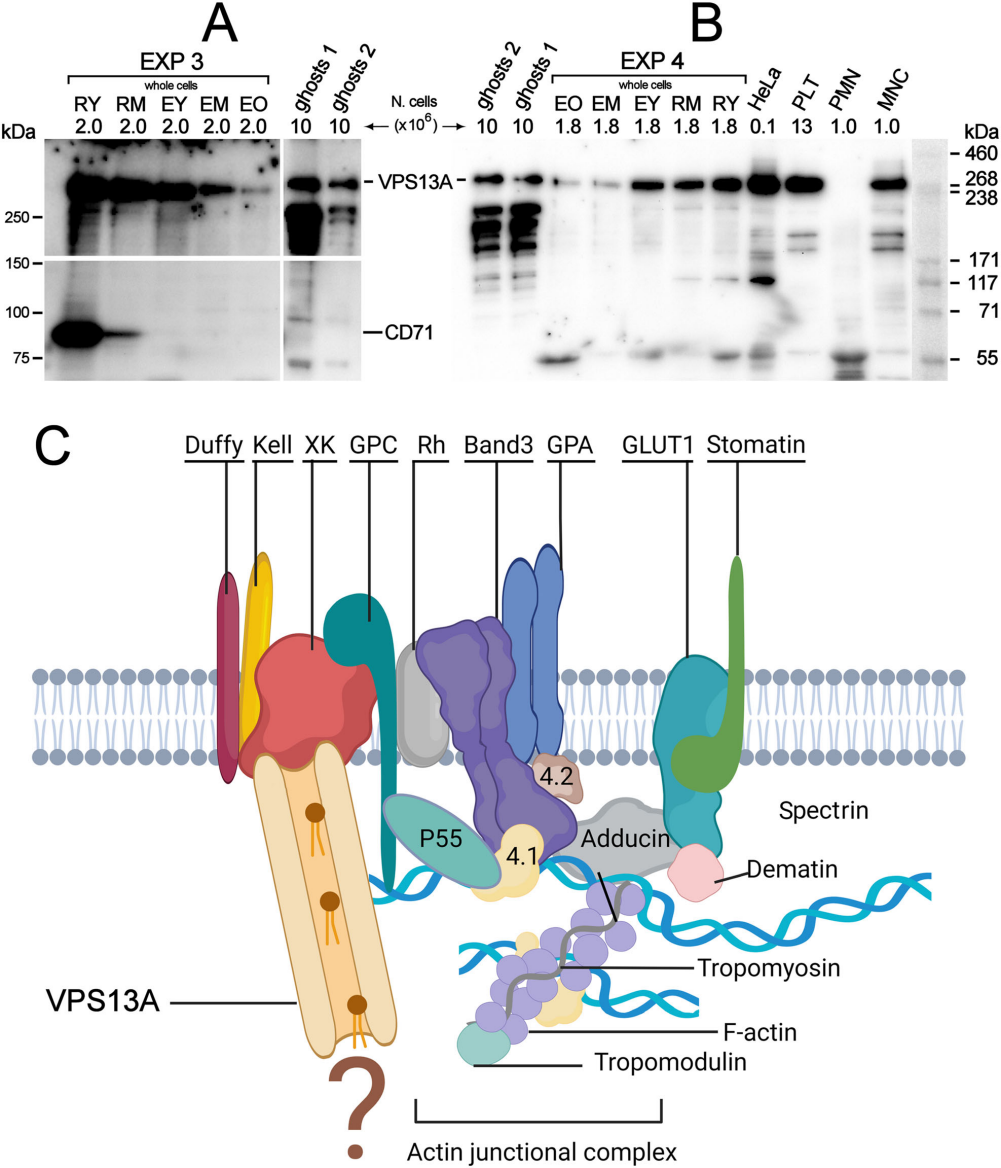
VPS13A in reticulocytes, RBCs and other cell types and its hypotetical disposition in the RBC membrane. **A** Western blotting of VPS13A of reticulocytes RY and RM, and RBCs of different age (EY, EM, EO). Reticulocytes and RBCs from EXP 3 were loaded as equal numbers (2.0 × 10^6^) of whole cells dissolved in sample buffer. Two samples of ghosts from two other different normal subjects were also loaded (ghost 1 and ghost 2, 10 × 10^6^ ghosts each), for comparison because this was the only sample form in which VPS13A was analysed in the literature. The lower panel shows the same membrane re-probed for CD71. **B** Western blotting for the detection of VPS13A in reticulocytes RY, RM, and RBCs of different age from another experiment. Polymorphonuclear cells, mononuclear cells and platelets freshly isolated from a normal human subject, and ghosts prepared from blood of two other independent subjects were also loaded in the amounts indicated. Finally, a sample of HeLa cells was loaded to allow comparison with the original work where detection of VPS13A (chorein) in RBC ghosts was first demonstrated and HeLa cells were used as a positive control [64]. **C** Schematic representation of the current model of the actin junctional complex in the RBC membrane [41] and the possible disposition of VPS13A in the membrane as deduced from published data.

Results shown in Fig. 7A, B confirm the presence of VPS13A in human reticulocytes and RBCs, and exclude a contribution from contaminating white cells or platelets. Although VPS13A displays a progressive and continuous decrease from the reticulocytes to old RBCs (17^th^ week), it remains in the membrane at significant levels at least until the mature RBC stage, contrary to CD71 that is selectively and completely lost within the first week of circulatory life of the cell.

## Discussion

The heterogeneity of young red cells is a key aspect explored in this study. Unique to our research is the isolation of two distinct subpopulations of circulating reticulocytes: young RY and older RM, and three subpopulations of RBCs of different age: EY, EM, EO. The isolated reticulocytes were “normal”, devoid of contamination by “stress” or “shift” reticulocytes [9], as well as by mature RBCs, distinguishing them from previous studies. We estimated that RY represent reticulocytes in their 12–24 h post-egress from the bone marrow, while RM represent cells in the subsequent 24–48 h of circulation. The subset of EY cells is indicative of RBCs in their first week of life. Conversely, EO cells correspond to RBCs in their 17^th^ week of life. The results of lipid analysis and morphological characterization allow reassessing some commonly accepted concepts about reticulocytes and their maturation. As RBCs age in circulation, they undergo coordinated membrane area and volume reduction to maintain a constant surface-to-volume ratio and prevent premature clearance due to spherization with consequent loss of deformability [23–27]. It is commonly believed that the cell-age related membrane loss occurs through the spontaneous release of spectrin-free vesicles [28, 29]. On the other hand, loss of membrane by early reticulocytes maturing in the bone marrow was described to occur via the release of exosomes enriched in the transferrin receptor (TfR, or CD71), and free of Band 3, glycophorin A and spectrin [17, 30]. However, we show here that circulating reticulocytes lose Band 3 and spectrin as they mature to EY (a process that will continue throughout the RBC’s 120-day circulatory lifespn [31].) suggesting that a significant difference exists between the bone marrow and circulatory phases of reticulocyte maturation [32].

The timing for development of a mature RBC should be extended from the conventional 1–2 days in the circulation, to at least one week, as judged from lipid composition. Achievement of full maturation could be evaluated, for instance, based on the inversion of the ratio between the relative amounts of palmitoyl-oleoyl (16:0/18:1) and of palmitoyl-linoleoyl (16:0/18:2) species of PC, which changes from >2 in reticulocytes to <1 in mature RBCs (Fig. 4 and Table S7). Furthermore, the results support the idea that circulating reticulocytes already exhibit a biconcave discocyte morphology [10, 11, 33], in contrast to a certain more diffuse perception that these cells may appear roundish, irregularly shaped, or multi-lobular [10, 34].

The present results confirm and extend previous evidence obtained by us that the increased relative levels of cholesterol and SM in relation to total lipids will produce a higher degree of liquid-ordered state, typical of membrane rafts, in the membrane of mature RBCs compared to that of the reticulocyte [35], potentially influencing membrane functionality, for instance the activity of membrane transport systems that is sensitive to the surrounding lipids [36–40].

We have shown previously that membrane rafts appear to be tightly anchored to the spectrin membrane skeleton in RBCs, through electrostatic interactions not involving the “canonical” Band 3-ankyrin and actin-protein 4.1-p55-GPC junctional complexes [35, 41]. The driving force for the selective retention of raft lipids could be the advantage of strengthening the interaction of the membrane skeleton with the bilayer through diffuse, direct interactions of phospholipids with spectrin. PE is a likely candidate for such interaction [42, 43]. We have examined in detail the diacyl species of PE, which amount to ≈49% of all PE, the rest being plasmalogens (alkylacyl ≈3%; alkenylacyl ≈48%) in human RBCs [20, 21]. Although detailed characterization of PE plasmalogens is in progress, it can be assumed that diacyl and alkenylacyl PE species change in unison during reticulocyte maturation. In fact, a correlation exists between the amounts of a given plasmenyl PE subspecies and the corresponding diacyl PE subspecies, indicating the common metabolic origin. Moreover major PE reorganization in the cell membrane due to exchange with plasma PE can be excluded because of the extremely low levels of PE in plasma lipoproteins [21, 44]. It is remarkable that, in front of considerable variability, in RBCs from different mammals, in the relative amounts of other phospholipids, PE displays the lowest variability of all [36] (Table S8). Taking a fresh perspective on the extensively studied topic of membrane skeleton biogenesis [45, 46], we suggest that the transformation of a relatively large discocytic reticulocyte into a smaller biconcave discocyte is primarily driven by a controlled reduction in cell volume and the loss of specific bilayer regions, rather than solely by the assembly of the spectrin skeleton. The discoid shape does not inherently signify the presence of a fully assembled membrane skeleton [47], as additional steps are required for its complete maturation from the already available full set of membrane skeletal components [48]. The relative instability of reticulocytes compared to mature RBCs can be attributed to the skeletal network still being somewhat “threadbare,” although it may already be “pinned” to the cytoplasmic leaflet by the interaction of spectrin with PE [49]. Early observations indicating that newly synthesized spectrin attaches to the plasma membrane through interactions distinct from spectrin-ankyrin and spectrin-protein 4.1, preceding its assembly into an extended meshwork [48, 50, 51], align perfectly with this model. This unique positioning of spectrin results in a “reduction of dimensionality” [52], thereby enhancing the rate of membrane skeleton assembly as the cell concurrently undergoes a reduction in volume and membrane area. The establishment of horizontal interactions and the anchoring to the bilayer via protein-protein vertical interactions could be considered complete only in mature RBCs [53].

The observed changes in membrane lipid composition can be most intuitively explained by the selective removal of certain lipids (PC, LPC, PI, and PS), that could be “pinched off” by macrophages, thereby leaving the remaining lipids either at constant levels (PE) or relatively enriched (SM and cholesterol), in a membrane that undergoes continuous reduction in area throughout reticulocyte maturation and beyond [32]. However, other processes could contribute to the observed lipid remodelling but, because circulating reticulocytes have lost the internal membranes on which phospholipid metabolism takes place [19, 32, 54], for instance the Kennedy’s pathways [55, 56], an exchange of lipids with the extracellular space must be invoked. RBCs are known to be able to exchange entire phospholipid molecules between the membrane and plasma [57, 58], the source of lipids being plasma lipoproteins. Moreover, some phospholipid classes can be translocated across the bilayer and remodelled in their acyl chains through the Lands cycle [59, 60]. To account for changes in subclasses of phospholipids of the inner leaflet, which cannot directly exchange with plasma, alternative mechanisms need to be considered [61, 62]. Amidst this uncertainty, what appears reasonably certain is that reticulocytes (and RBCs) must depend on external conditioning, not necessarily involving only splenic macrophages [63]. These agents and the mechanistic details by which they operate still have to be identified. Lipid exchange with the surrounding environment could be mediated by lipid transfer proteins. VPS13A, previously known as chorein, is a large protein found ubiquitously in normal tissues that belongs to a family of bridge-like lipid transfer proteins involved in non-vesicular lipid trafficking within eukaryotic cells, facilitating lipid exchange at membrane contact sites between subcellular organelles and between organelles and the plasma membrane [16]. Mutations in the *VPS13A* gene result in either the absence or significantly reduced expression of the protein in all tissues, including RBCs, in patients with Chorea acanthocytosis [64], where loss of VPS13A function not only contributes to neurological symptoms but also leads to abnormal RBC morphology. This abnormality may arise early in erythropoiesis due to impaired lipid trafficking in nucleated erythroid precursors and persist as a distinct feature of mature acanthocytes. However, VPS13A might play a crucial role in lipid remodelling also in circulating reticulocytes and RBCs, where it is allegedly still present at high levels. Because expression of VPS13A in RBCs was documented by immunoblotting of samples of ghosts prepared from whole blood that was previously frozen and then thawed, [64, 65], concerns could be raised about possible artefactual results [66]. However, we have confirmed here that the protein is mostly expressed in RBC among blood cells. It declines as reticulocytes and young RBCs mature, but it is still present at significant levels in mature RBCs. The fact that 5 to 10 times less protein seems to be present when ghosts are analysed instead of whole RBCs deserves further investigation. It may be related to losses incurred during ghost preparation, because, for instance, part of the protein may be located in the cytoplasm or only loosely associated to the membrane. VPS13A interactions with membrane proteins have been described in the literature, in particular with XK, β-adducin and β-actin [67, 68]. It is intriguing that a protein facilitating lipid exchange at contact sites between membranes of intracellular organelles remains unaffected by the gradual breakdown of unnecessary components typical of reticulocyte maturation and it is found in significant quantities in mature RBCs, which lack internal membranes. It has been recently shown that mice with conditionally knocked down VPS13A display an increased number of immature reticulocyte in the circulation [69]. One potential avenue for elucidating this phenomenon lies in exploring the interaction between VPS13A and the scramblase XK [70] as it is conceivable that VPS13A could supply the lipids necessary for XK-mediated scrambling between the two leaflets of the membrane, thereby facilitating coordinated changes in the composition of these leaflets. However, it remains a mystery how VPS13A could act according to this mechanism in a cell type that is lacking internal membranes from where to derive the required lipids [71].

The results obtained may have significant implications for several unresolved issues in RBC biology. The intricate process of lipid remodelling in reticulocytes preceding their transition into functional circulating RBCs warrants further exploration to uncover its underlying mechanisms. This inquiry holds broad significance, particularly in elucidating protein-lipid interactions pertinent to membrane transport systems and membrane skeleton biogenesis. Understanding the possible role of lipid transfer proteins in facilitating lipid exchange between reticulocytes/RBCs and their environment is crucial. Identifying the key regulators of reticulocyte maturation in the bloodstream holds promise for generating fully mature RBCs in vitro for transfusion purposes. While the findings presented here do not offer a precise sequence of events leading to the observed differences, they illuminate several potential avenues for future research in this domain.

## Materials and methods

A detailed version of Materials and Methods is available in the Supplementary material. Human blood samples were collected in lithium heparin as the anticoagulant at the local Transfusion center (Servizio di Immunoematologia e Medicina Trasfusionale of the IRCCS Policlinico San Matteo, Pavia, Italy) from regular donors after informed consent was obtained (protocol approved on 2017/04/10, by the local ethics committee: Comitato Etico Area Pavia, IRCCS Policlinico San Matteo, Pavia, Italy). Blood was leukodepleted and RBCs suspended at ≈20% haematocrit (Ht) in PBSG (5 mM sodium phosphate pH 7.4, 154 mM NaCl, 4.5 mM KCl, 305–310 mosmol/kg H_2_O). Aliquots were used for cell count, Ht and Hb determination (Drabkin’s reagent), SDS-PAGE and Western blotting, fixation for SEM, and lipid extraction for lipidomics analysis. The RBC suspension was then used for the immunomagnetic isolation of CD71^+^ reticulocytes by using the “CD71 MicroBeads” system (Miltenyi Biotec, Germany). By modulating the flow rate of the RBC suspension passing through the magnetized separation columns, two different populations of reticulocytes could be isolated, and named RY, for “young reticulocytes” and RM for more “mature reticulocytes”.

### Separation of RBCs into subpopulations of different age

After isolation of CD71^+^ reticulocytes, the reticulocyte-depleted RBC suspension was subjected to centrifugation in self-forming Percoll^®^ Plus (GE Healthcare, Milan, Italy) gradients according to a previously published method [72], with minor modifications (see Supplementary material). Three subpopulations of cells of different density were isolated from the gradients: young erythrocytes (EY), erythrocytes of middle-age (EM) and old erythrocytes (EO).

### Preparation of cells for scanning electron microscopy (SEM)

RBCs and reticulocytes were fixed with glutaraldehyde (GA) according to a two-step protocol [73]. Fifty μl of a suspension in water of GA-fixed cells were mixed with 50 μl of 2% osmium tetroxide in water (Cod. 75632-5 ML, Merck Life Science S.r.l., Milan, Italy). After 30 min at room temperature, cells were washed with deionized water and were subjected to graded alcohol treatment, starting from 200 μl 70% ethanol, followed after 30 min by 80% ethanol and, after another 30 min, by absolute ethanol, in which cells could be stored at 4 °C. For SEM, a few μl of these suspensions were poured on the surface of a 5 mm × 7 mm silicon chip (Ted Pella Inc, Redding, CA, USA). After evaporation of the solvent, the chip was laid on one side of a bi-adhesive plastic disc coated with conductive material (Ted Pella Inc, Redding, CA, USA) which was attached to the surface of a standard pin stub mount for SEM and made electrically conductive by a coating of Pt using a Cressington 208HR Sputter Coater. SEM images were obtained through a FEG-SEM TESCAN Mira3 XMU, Variable Pressure Field Emission Scanning Electron Microscope (TESCAN, Brno, Czech Republic) located at the Arvedi Laboratory, CISRiC, Pavia, Italy. Observations were made, at different magnifications, in secondary electrons mode at 20 kV with an In-Beam SE detector at a working distance of 5 mm.

### Lipid analysis

Aliquots of packed reticulocytes or RBCs from four independent donors, containing 2 × 10^7^ cells, were extracted with 1 ml of ice-cold methanol and transferred to 10 ml Pyrex^®^ glass tubes with polyphenolic screw cap. The quantitative lipid measurement was done by high-resolution mass spectrometry (LTQ-Orbitrap, Thermo Scientific) [74]. Lipids were extracted with an established Methyl-tert-butylether protocol [75]. The Orbitrap Velos Pro hybrid mass spectrometer was operated in Data Dependent Acquisition mode using a HESI II ion source. Full scan profile spectra from m/z 450 to 1050 for positive ion mode and from m/z 400 to 1000 for negative ion mode were acquired in the Orbitrap mass analyser at a resolution of 100k at m/z 400 and <2 ppm mass accuracy. Samples were measured once in positive polarity and once in negative polarity. For MS/MS experiments, the 10 most abundant ions of the full scan spectrum were sequentially fragmented in the ion trap using He as collision gas (CID, Normalized Collision Energy: 50; Isolation Width: 1.5; Activation Q: 0.2; and Activation Time: 10). Centroided product spectra at a normal scan rate (33 kDa/s) were collected. The custom developed software tool Lipid Data Analyzer was used for data analysis [76]. Seven classes of lipids were quantified, together with their subclasses according to the length and number of double bonds of the acyl chains linked to the glycerol or sphingosine moiety: phosphatidylcholine (PC), lysophosphatidylcholine (LPC), sphingomyelin (SM), phosphatidylethanolamine (PE), phosphatidylserine (PS), phosphatidylinositol (PI) and cholesterol (Chol). The low amounts of reticulocytes that could be isolated restricted the lipidomics characterization to direct MS analysis without intermediate chemical steps, providing only limited information on the chemical structure of each phospholipid species [77]. Some of the missing information was recovered by annotating most possible phospholipid subclasses based on previously published characterizations of the human RBC lipidome [20].

### SDS-PAGE and Western blotting

Standard protocols were adopted for SDS-PAGE in 10% isocratic or 5–15% gradient polyacrylamide mini-gels (Bio-Rad Laboratories S.r.l., Segrate, Italy), and for Western blotting by electro-transferring proteins from gels to PVDF membranes (0.2 μm pores) using a Trans Blot Turbo system (Bio-Rad Laboratories S.r.l., Segrate, Italy) according to manufacturer’s instructions. After incubation with the primary antibody, and the appropriate secondary horseradish-peroxidase-(HRP)-conjugated antibody, membranes were developed with the chemiluminescence kit Prime Western Blotting Detection Reagent (GE Healthcare, Milan, Italy) and the signal was acquired with a Molecular Imager ChemiDoc XRS+ (Bio-Rad Laboratories S.r.l., Segrate, Italy). Antibodies usedwere: mouse monoclonal (BIII-136) anti human Band 3 (B9277, Merck Life Science S.r.l., Milan, Italy). Mouse monoclonal (H68.4) anti human CD71, sc-51829. Mouse monoclonal anti human β-spectrin (VD4), sc-53901 (Santa Cruz Biotechnology, Dallas, Texas, USA). Rabbit polyclonal anti human VPS13A (28618-1-AP, Proteintech Germany GmbH, Planegg-Martinsried, Germany). Secondary antibodies used were: HRP-conjugated goat anti-mouse IgGs (170-6516) and HRP-conjugated goat anti-rabbit IgGs (170-6515) (Bio-Rad Laboratories S.r.l., Segrate, Italy).

### Statistics

Statistical analysis was performed with Prism (GraphPad Software, Boston, MA, U.S.A.). Individual tests applied to the data are indicated in the text.

## Supplementary information

Supplemental material

original Western blottings

additional original western blottings

## Data Availability

The materials described in the article, including all relevant raw data, will be made available by the authors upon request for non-commercial purposes.
