# Supplementary material for: Insights from lipidomics into the terminal maturation of circulating human reticulocytes
Source: Cell Death Discov. 2025 Feb 27;11:79. doi: 10.1038/s41420-025-02318-x (PMC11868425; doi:10.1038/s41420-025-02318-x)

Original Western blotting for CD71 as displayed in Figure 1A of the manuscript. Before blotting, the membrane was cut in two halves. The upper part was probed for spectrin, the bottom part for CD71. The left part of the figure (seven lanes including the markers) is related to the single experiment reported in Figure 1A.

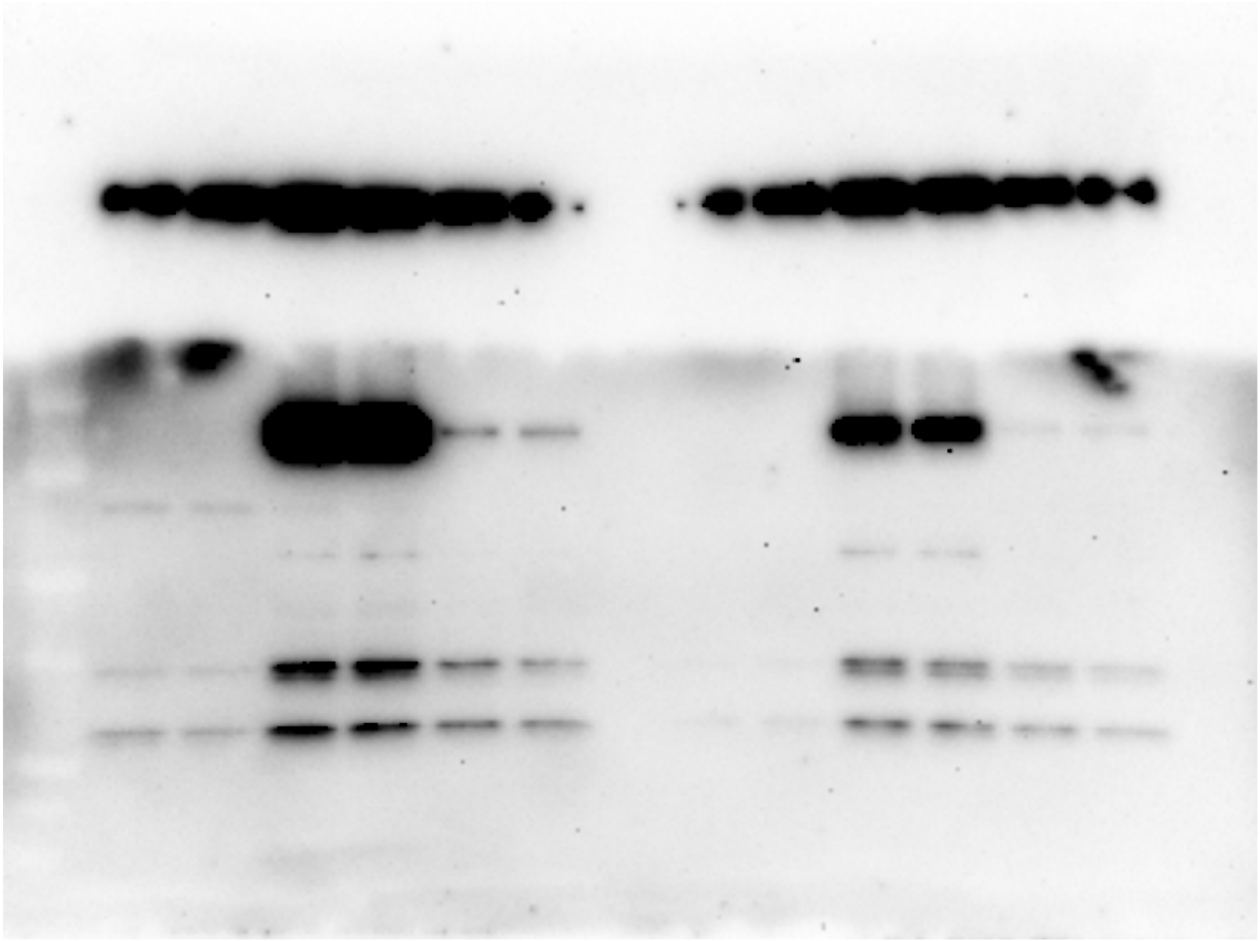

Original Western blotting for protein 4.1R as shown in Figure1B of the manuscript for which, the first four lanes are used, related to a single experiment (EXP3).

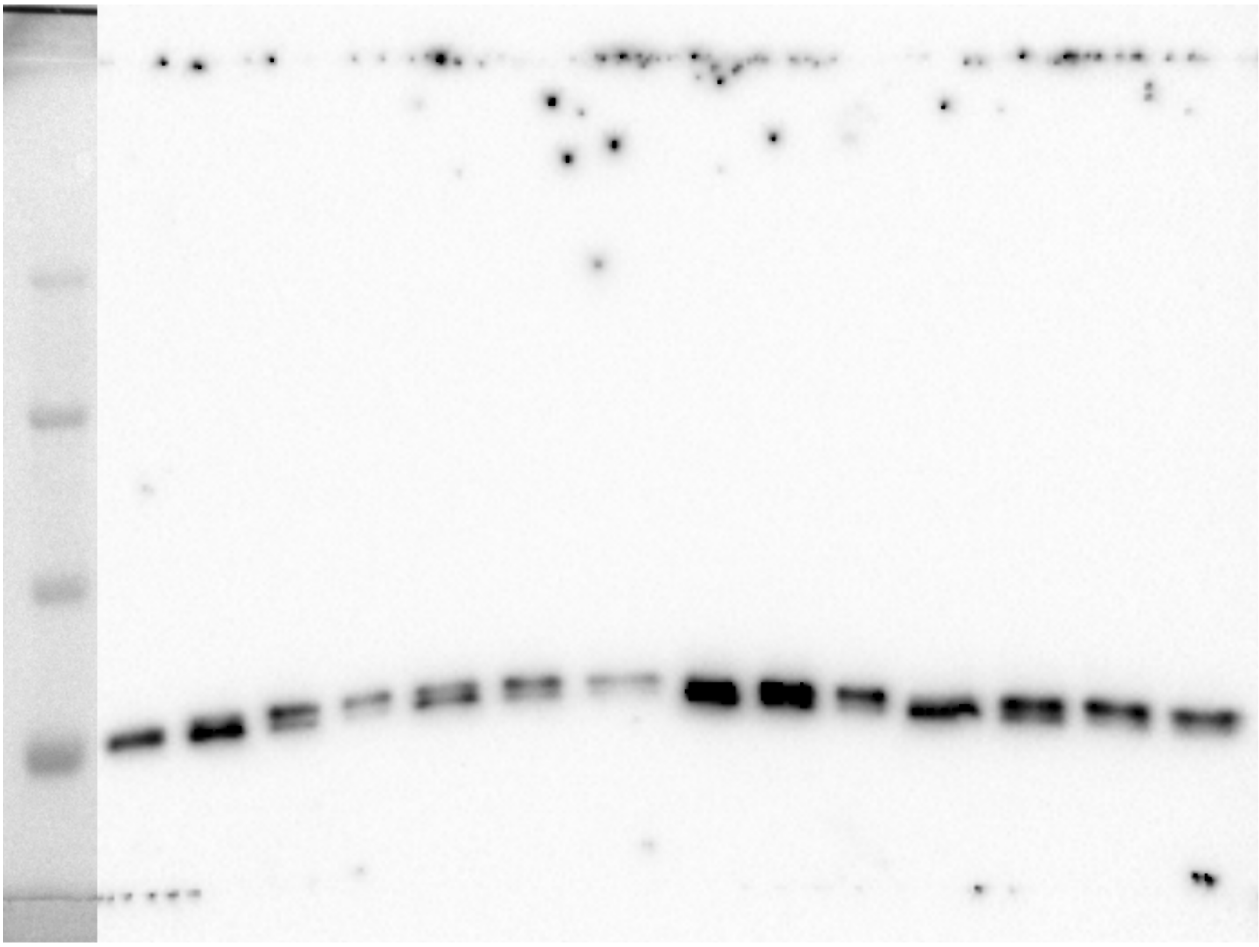

Original Western blotting for protein 4.1R as shown in Figure1D, EXP1 of the manuscript, for which the lanes 8, 9 and 10 of the blot shown below were used.

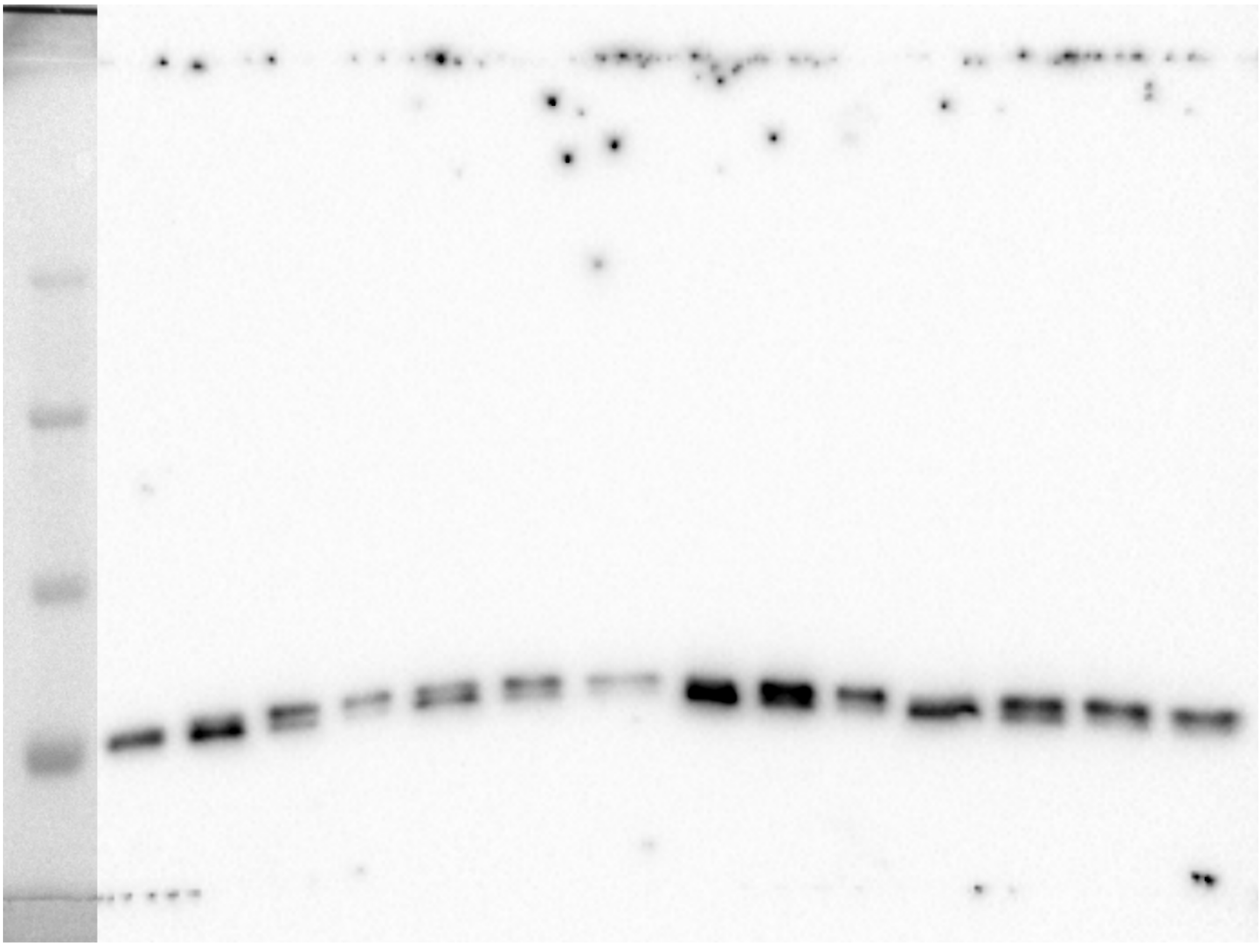

Original Western blotting for protein 4.1R as shown in Figure1D, EXP2 and EXP4, corresponding, respectively, to lanes 5, 6, 7 and 11, 12, 13 of the original blotting shown here.

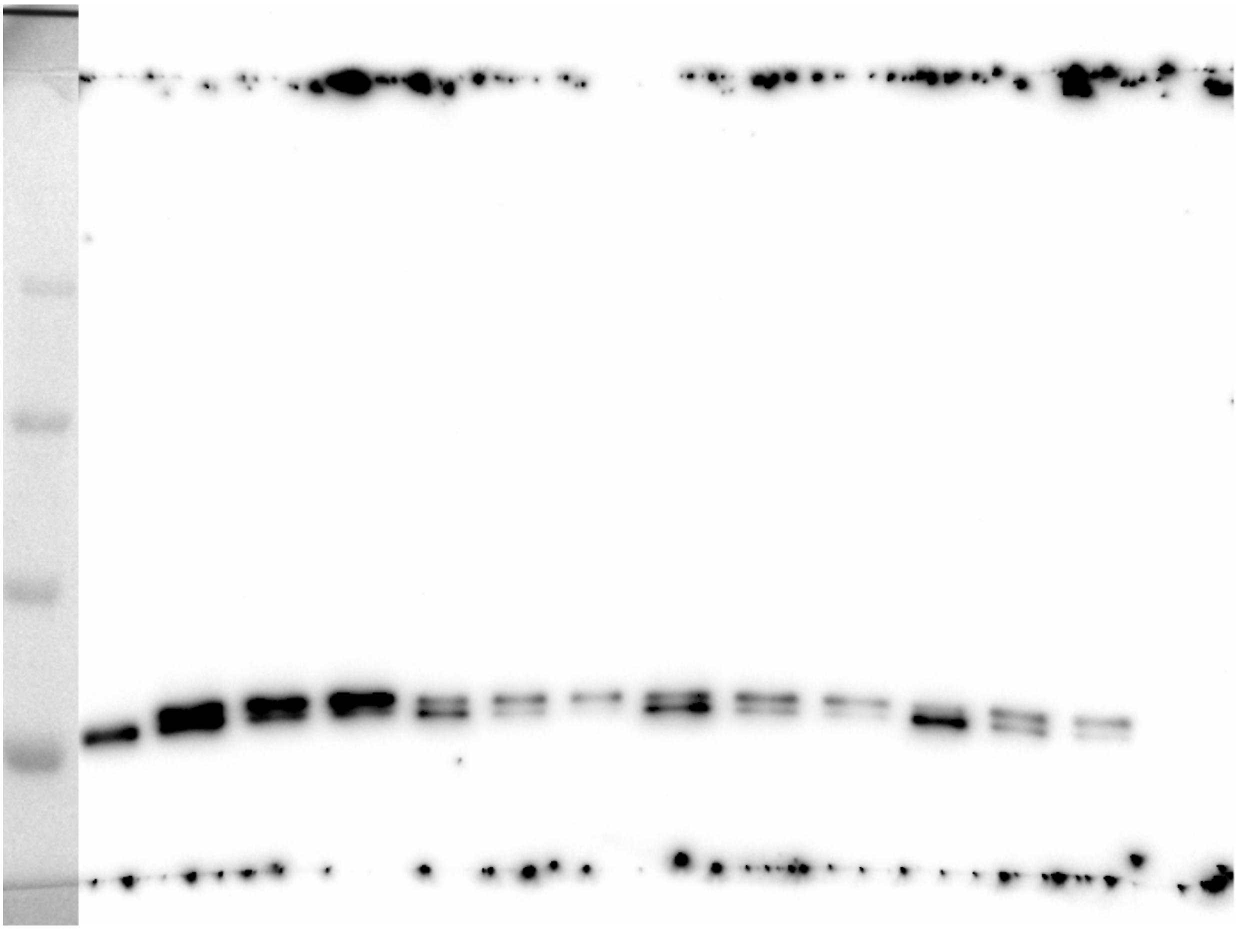

Original Western blotting for protein 4.1R as shown in Figure1D, EXP3 of the manuscript, for which the lanes 1, 2 and 3 of the blot shown below were used.

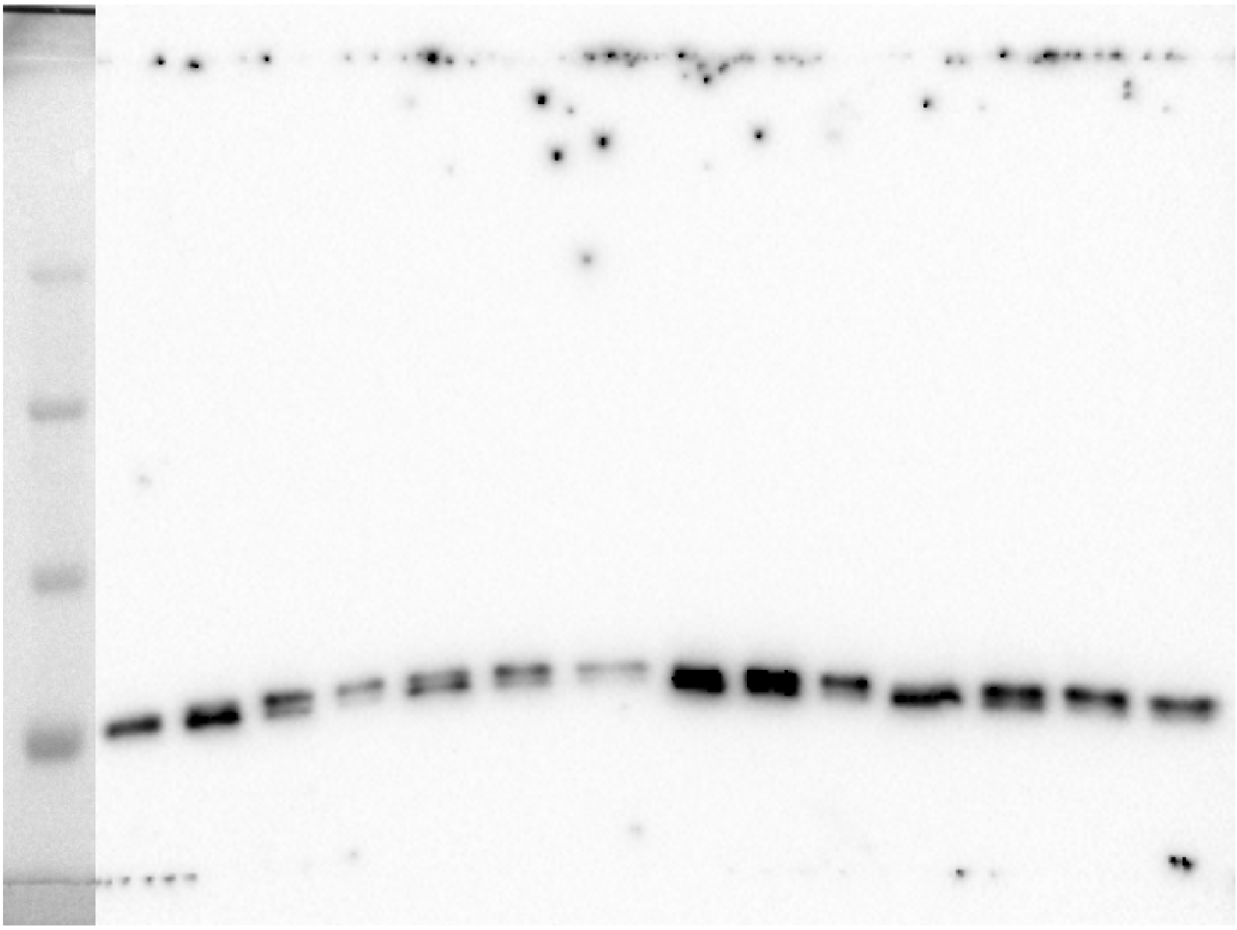

Original Western blotting for β-spectrin as shown in Figure1E, EXP6 of the manuscript. Original Western blotting for CD71 as displayed in Figure 1A of the manuscript. Before blotting, the membrane was cut in two halves. The upper part was probed for β-spectrin (shown here), the bottom part for Band 3. The left part of the figure (six lanes) is related to the single experiment reported in Figure 1E (EXP6).

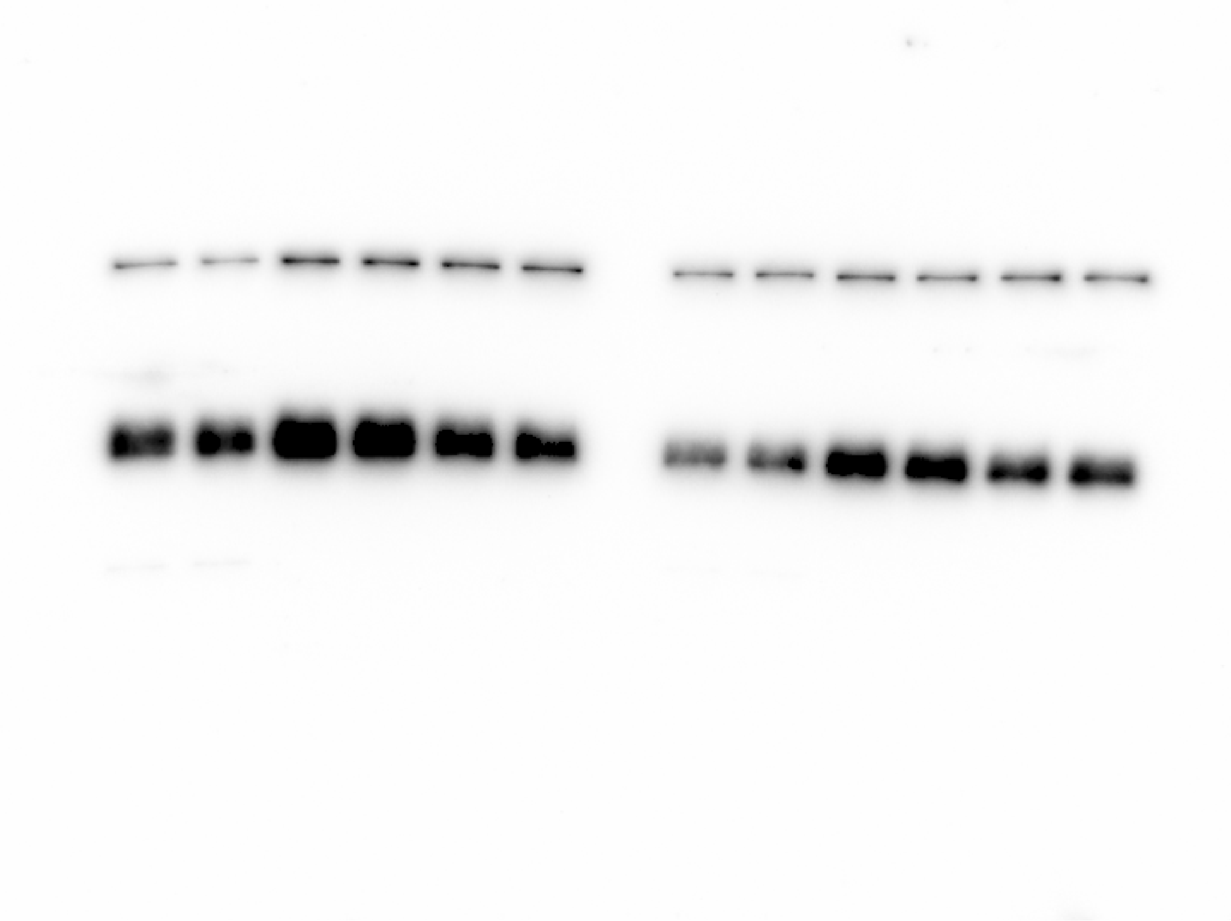

Original Western blotting for β-spectrin as shown in Figure1E, EXP6 of the manuscript. Before blotting, the membrane was cut in two halves. The upper part was probed for β-spectrin, the bottom part for Band 3. The left part of the figure (six lanes) is related to the single experiment reported in Figure 1E (EXP6). This is a different exposure of the same membrane shown in the previous page, for a better visualization of Band 3 in the various samples.

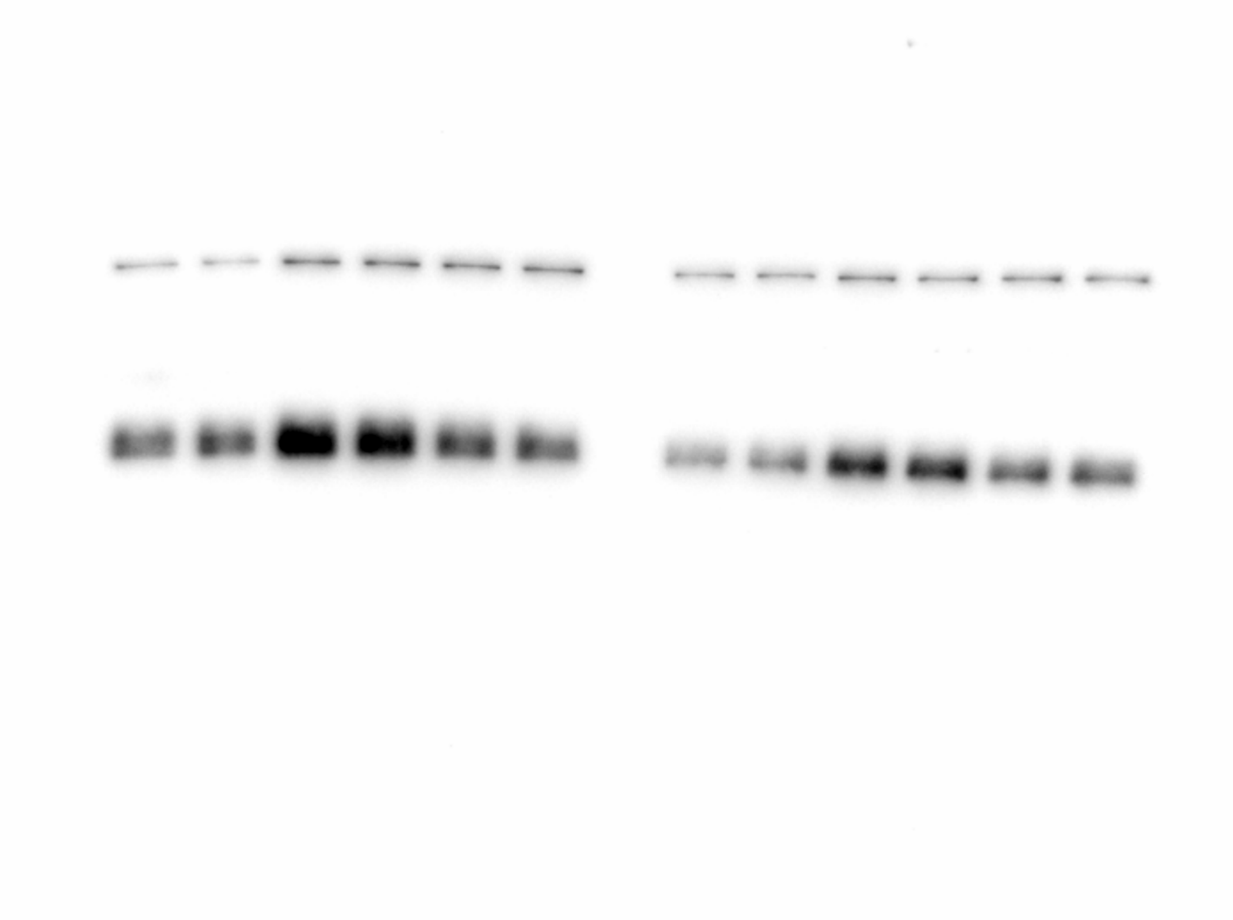

Original Western blotting for VPS13A Figure7A, upper panel, of the manuscript. After probing for VPS13A the same membrane was probed with anti CD71 (see next page).

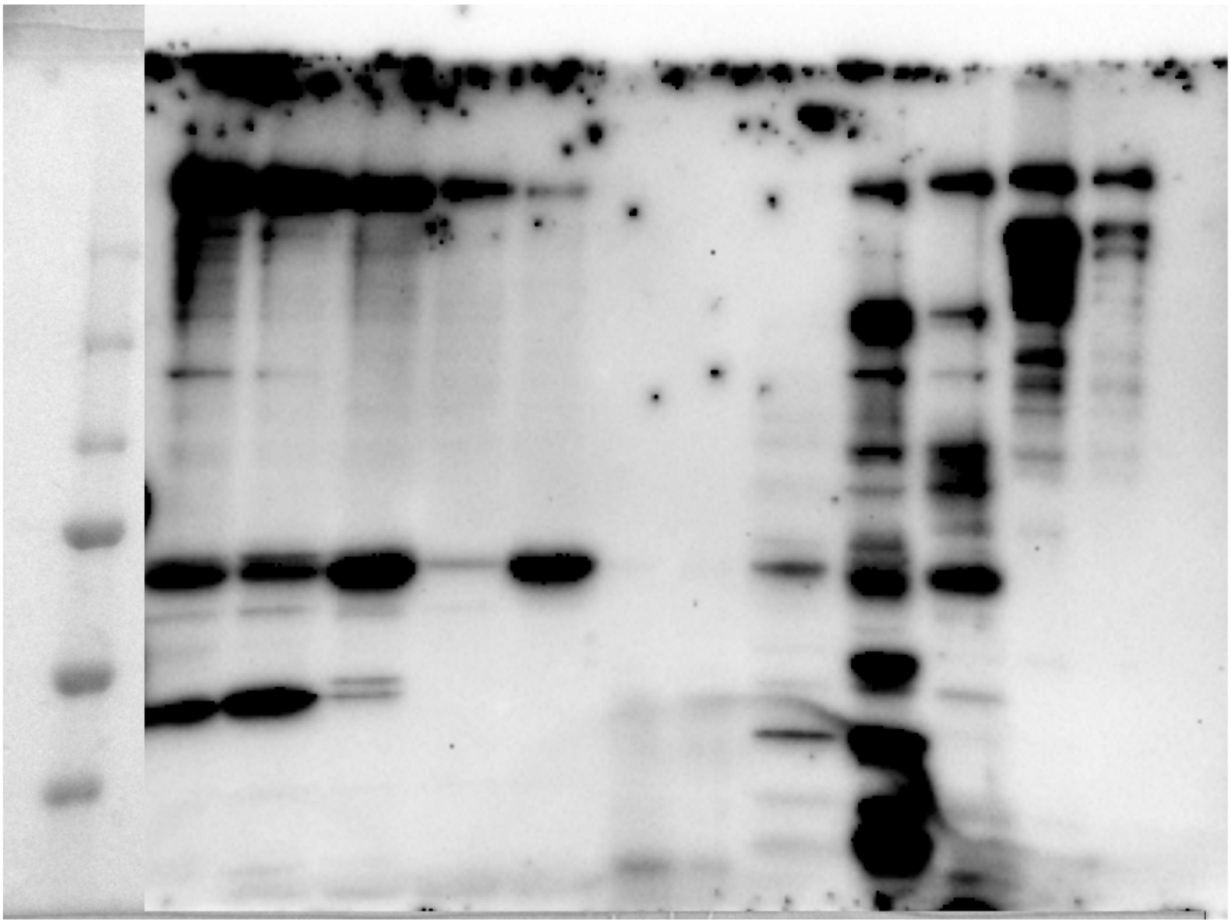

Original Western blotting for CD71 Figure7A, lower panel, of the manuscript. Before probing for CD71 the same membrane was probed for VPS13A (see previous page).

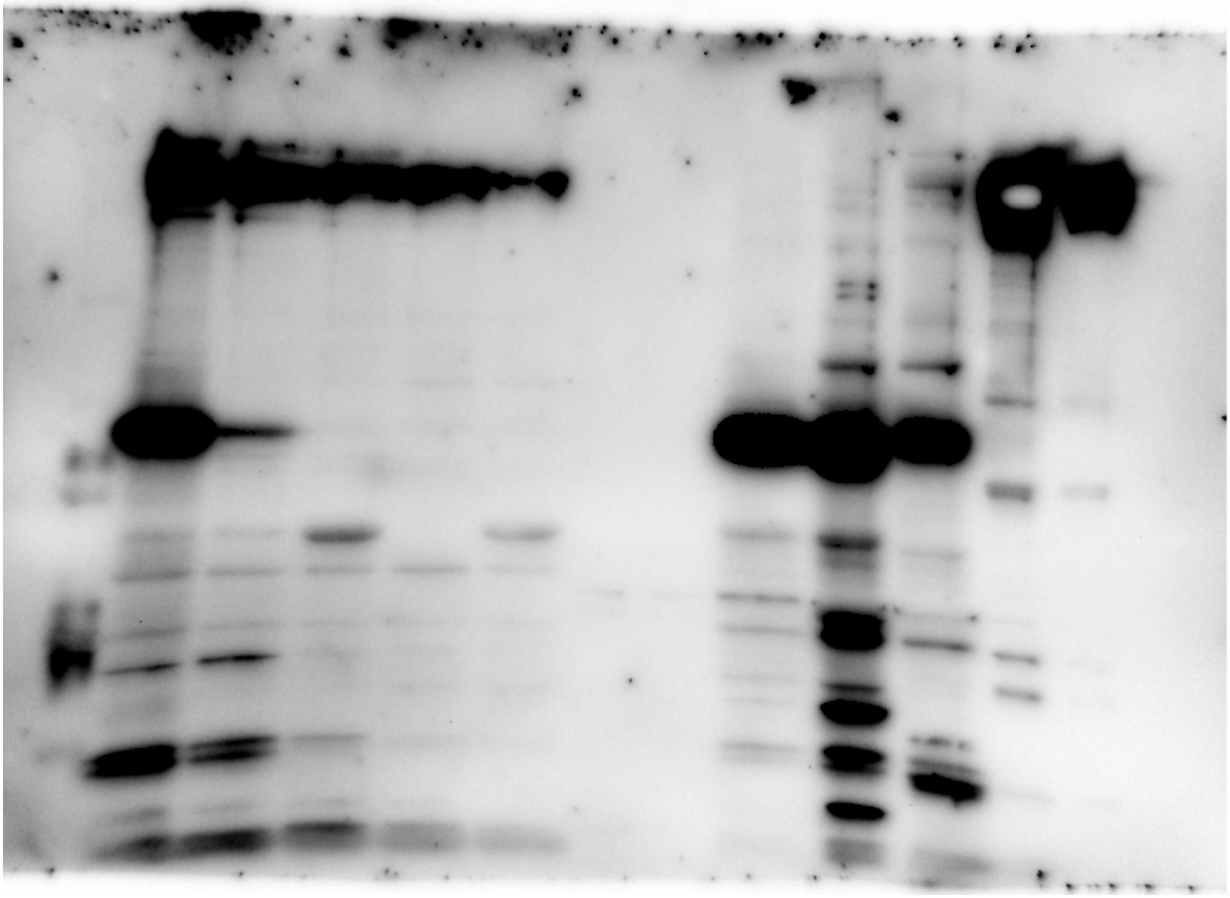

Supplement: Supplementary file 2 — original Western blottings [file 41420_2025_2318_MOESM2_ESM.pdf]
