# Supplementary material for: Insights from lipidomics into the terminal maturation of circulating human reticulocytes
Source: Cell Death Discov. 2025 Feb 27;11:79. doi: 10.1038/s41420-025-02318-x (PMC11868425; doi:10.1038/s41420-025-02318-x)
Supplement: Supplementary file 3 — additional original western blottings [file 41420_2025_2318_MOESM3_ESM.pdf]

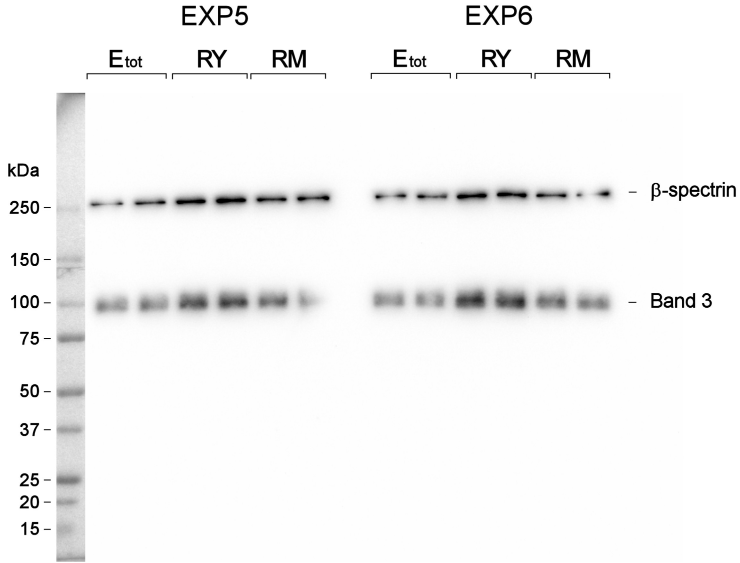
EXP5
EXP6
Etot
RY
RM
Etot
RY
RM
kDa
250
150
100
75
50
37
25
20
15
β-spectrin
Band 3

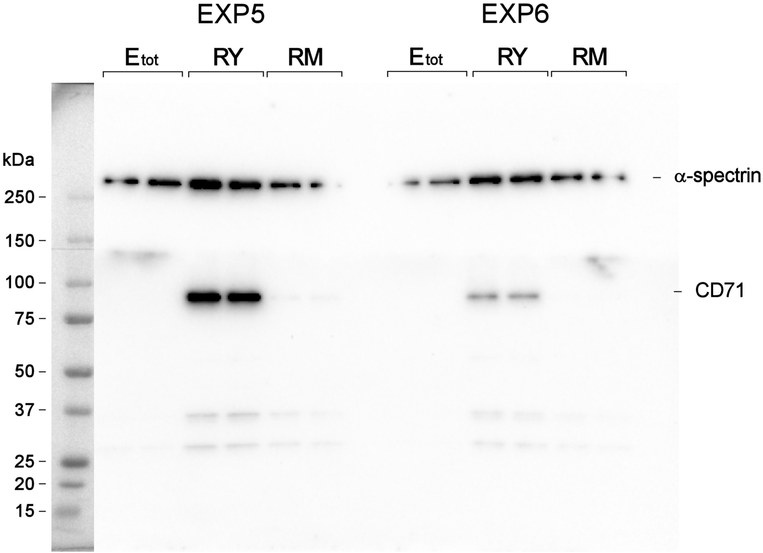
EXP5
EXP6
Etot
RY
RM
Etot
RY
RM
kDa
250
150
100
75
50
37
25
20
15
α-spectrin
CD71

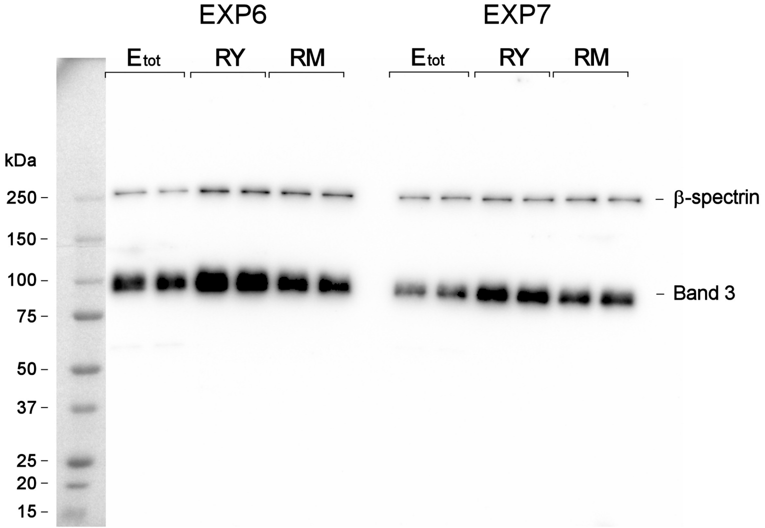
EXP6
EXP7
Etot
RY
RM
Etot
RY
RM
kDa
250
150
100
75
50
37
25
20
15
β-spectrin
Band 3

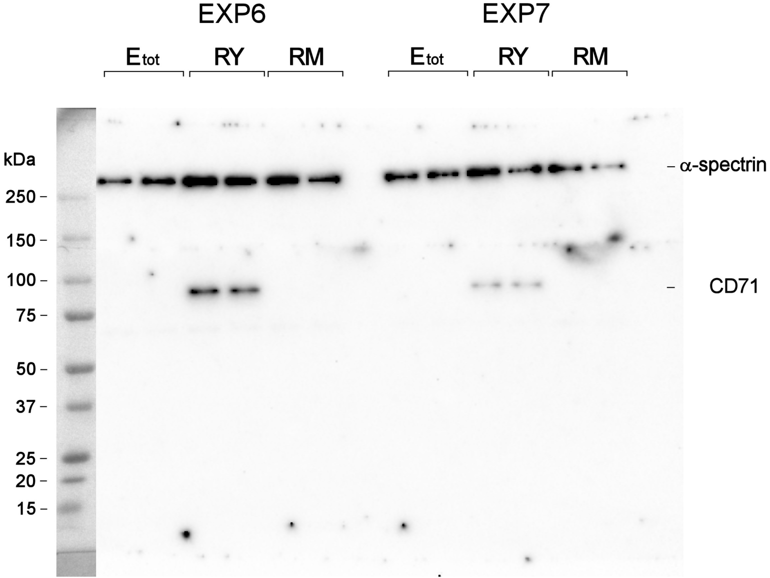

EXP6
EXP7
Etot
RY
RM
Etot
RY
RM
kDa
250
150
100
75
50
37
25
20
15
α-spectrin
CD71

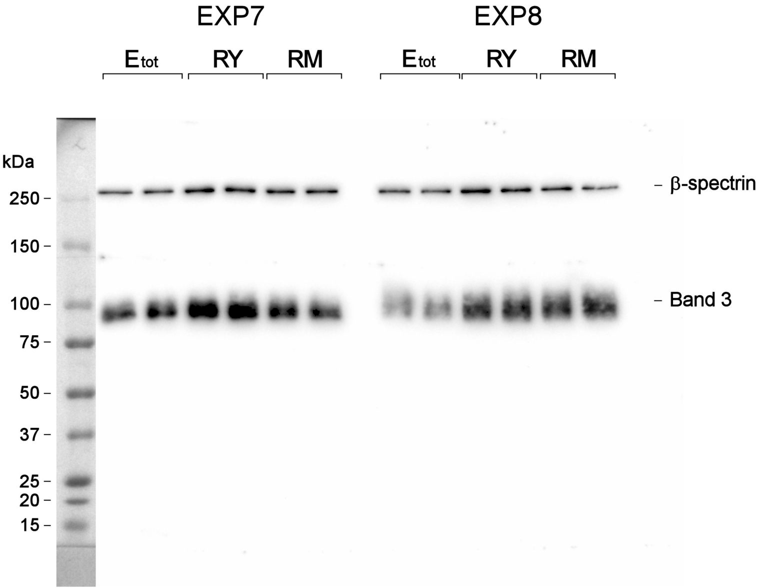
EXP7
EXP8
Etot
RY
RM
Etot
RY
RM
kDa
250
150
100
75
50
37
25
20
15
β-spectrin
Band 3
